# Expression profiling of the adhesion G protein-coupled receptor GPR133 (ADGRD1) in glioma subtypes

**DOI:** 10.1093/noajnl/vdaa053

**Published:** 2020-04-28

**Authors:** Joshua D Frenster, Michael Kader, Scott Kamen, James Sun, Luis Chiriboga, Jonathan Serrano, Devin Bready, Danielle Golub, Niklas Ravn-Boess, Gabriele Stephan, Andrew S Chi, Sylvia C Kurz, Rajan Jain, Christopher Y Park, David Fenyo, Ines Liebscher, Torsten Schöneberg, Giselle Wiggin, Robert Newman, Matt Barnes, John K Dickson, Douglas J MacNeil, Xinyan Huang, Nadim Shohdy, Matija Snuderl, David Zagzag, Dimitris G Placantonakis

**Affiliations:** 1 Departments of Neurosurgery, New York, New York, USA; 2 Pathology, New York, New York, USA; 3 Neurology, New York, New York, USA; 4 Radiology, New York, New York, USA; 5 Biochemistry and Molecular Pharmacology, New York, New York, USA; 6 NYU Grossman School of Medicine, New York, New York, USA; Kimmel Center for Stem Cell Biology, NYU Grossman School of Medicine, New York, New York, USA; 7 Laura and Isaac Perlmutter Cancer Center, NYU Grossman School of Medicine, New York, New York, USA; 8 Brain and Spine Tumor Center, NYU Grossman School of Medicine, New York, New York, USA; 9 Institute for Systems Genetics, NYU Grossman School of Medicine, New York, New York, USA; 10 Rudolf Schönheimer Institute of Biochemistry, Medical Faculty, University of Leipzig, Leipzig, Germany; 11 Heptares Therapeutics Ltd, Cambridge, UK; 12 AptaChem Consulting LLC, Apex, North Carolina, USA; 13 Office for Therapeutic Alliances, NYU Grossman School of Medicine, New York, New York, USA; 14 Neuroscience Institute, NYU Grossman School of Medicine, New York, New York, USA

**Keywords:** adhesion, glioblastoma, glioma, GPR133, G protein-coupled receptor

## Abstract

**Background:**

Glioma is a family of primary brain malignancies with limited treatment options and in need of novel therapies. We previously demonstrated that the adhesion G protein-coupled receptor GPR133 (ADGRD1) is necessary for tumor growth in adult glioblastoma, the most advanced malignancy within the glioma family. However, the expression pattern of GPR133 in other types of adult glioma is unknown.

**Methods:**

We used immunohistochemistry in tumor specimens and non-neoplastic cadaveric brain tissue to profile GPR133 expression in adult gliomas.

**Results:**

We show that GPR133 expression increases as a function of WHO grade and peaks in glioblastoma, where all tumors ubiquitously express it. Importantly, GPR133 is expressed within the tumor bulk, as well as in the brain-infiltrating tumor margin. Furthermore, GPR133 is expressed in both isocitrate dehydrogenase (IDH) wild-type and mutant gliomas, albeit at higher levels in IDH wild-type tumors.

**Conclusion:**

The fact that GPR133 is absent from non-neoplastic brain tissue but de novo expressed in glioma suggests that it may be exploited therapeutically.

**Key Points**

- GPR133 is absent in a healthy brain but de novo expressed in glioma.
- GPR133 expression increases as a function of WHO grade and is highest in glioblastoma.
- GPR133 is expressed in both the tumor core and its brain-infiltrating edge.
- GPR133 is expressed in both IDH wild-type and mutant gliomas, albeit at higher levels in the former.

**Importance of the Study**

Gliomas are refractory to current treatments. There is an incontestable need to identify new therapeutic targets. Our study indicates that GPR133, an adhesion G protein-coupled receptor, may represent an appealing new target, by virtue of being de novo expressed in glioma, while absent from non-neoplastic brain tissue. GPR133 expression increases with anaplasia and is highest in glioblastoma, where it is expressed in all tumors. Importantly, it is found not only within the tumor bulk, but also in the brain-infiltrating edge. The expression profile of GPR133, and our previous studies that indicate that GPR133 is necessary for glioblastoma growth, suggests that GPR133 may be pursued as a new treatment opportunity.

GPR133 (ADGRD1) is a member of the adhesion family of G protein-coupled receptors,^1–5^ whose function in health and disease is poorly understood. We recently reported that GPR133 is expressed in glioblastoma (GBM), the most common brain malignancy, while normal brain tissue has no baseline expression.^6,7^ Using patient-derived cultures and xenografts of GBM, we showed that knockdown of GPR133 impairs tumor initiation in vitro and in vivo, suggesting GPR133 supports tumor growth.^6^ Consistent with this hypothesis, increased *GPR133* mRNA transcript correlates with reduced patient survival in the TCGA database.^6^ These findings suggest GPR133 represents a novel therapeutic target in GBM.

Glioma comprises a heterogeneous group of primary brain malignancies with distinct genetic drivers, molecular markers, and clinical behaviors. The WHO has traditionally graded gliomas histologically as grades I–IV, with grade IV, GBM, being the most aggressive and most common diagnosis. However, over the past decade, the emergence of molecular markers has redefined the WHO classification.^8–14^ In adult gliomas, neomorphic mutations in isocitrate dehydrogenase 1 and 2 (*IDH1* and *IDH2*), most commonly R132H and R172K, respectively, are the most important molecular classifiers. *IDH* mutant gliomas are typically diagnosed in younger adults and further subclassified into 1p19q codeleted tumors, traditionally known as oligodendrogliomas, and 1p19q intact tumors, also referred to as astrocytomas. The latter commonly show loss of tumor suppressor *TP53* and loss-of-function mutations in the chromatin remodeler *ATRX* concurrently with the *IDH* mutation. *IDH* wild-type gliomas, on the other hand, are usually seen in older adults, are linked with a different set of genetic drivers, and carry the worst prognosis. Well-demarcated gliomas identified by mutations in oncogene *BRAF* are usually classified as grade I tumors histologically.

Our previously reported experimental observations were based on *IDH* wild-type GBM.^6^ However, the expression profile of GPR133 in other glioma subtypes is unknown. Here, we use a mouse monoclonal antibody against the N terminus of GPR133^6^ to investigate its expression in a cohort of archived glioma specimens from our institution. Our findings indicate that GPR133 is expressed in both *IDH* wild-type and mutant tumors of grades II–IV, but is not found in non-neoplastic brain tissue, including the subventricular zone that harbors neural progenitors thought to give rise to gliomas.^15^ In addition, GPR133 is expressed not only within the tumor bulk, but also in the brain-infiltrating tumor edge. These findings are consistent with our analysis of the TCGA dataset and suggest that GPR133 may be exploited therapeutically in a wide range of adult glioma subtypes.

## Materials and Methods

### Case Acquisition

Brain tumor samples were obtained retrospectively from 67 patients who underwent surgical resection since 2013 at New York University Langone Medical Center (NYULMC). All tumor specimens in this study became available from the NYU Pathology Department as formalin-fixed, paraffin-embedded (FFPE) tumor blocks and were de-identified of patient information. Control non-neoplastic brain samples were obtained from 5 autopsies and 1 resected temporal lobe from a patient undergoing epilepsy surgery.

We surveyed the pathology reports of these 67 patients for molecular markers used in glioma classification (Supplementary Table 1). These markers included immunohistochemistry (IHC) for the R132H variant of IDH1, TP53, ATRX, and V600E variant of BRAF; fluorescent in situ hybridization for epidermal growth factor receptor (*EGFR*) amplification; 1p19q status by loss of heterozygosity and PCR; and *MGMT* promoter methylation by methylation-specific PCR assay or pyrosequencing. In addition, we surveyed mutational results on 52 genes commonly mutated in cancer using the clinically validated targeted next-generation sequencing (NGS) Oncomine panel (ThermoFisher). This panel includes the *IDH1* and *IDH2* genes. Finally, we performed molecular subtyping of these tumors with the Illumina 450K or EPIC DNA methylation arrays,^16,17^ which is clinically validated at NYULMC, as previously described.^18^ There were no H3 K27M mutant gliomas in our cohort.

Retrospective analysis of patient records was approved by NYULMC’s Institutional Review Board (IRB protocol 11-01733).

### Immunohistochemistry

**Figure 1.**
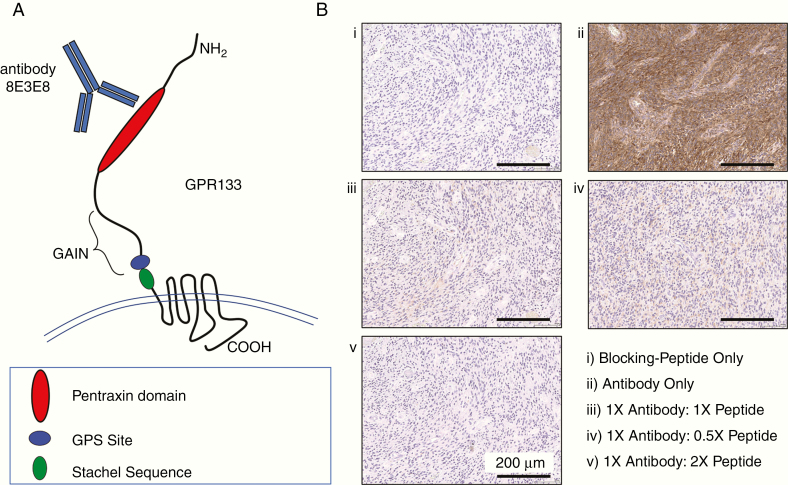
The mouse monoclonal antibody 8E3E8 detects the extracellular pentraxin domain of human GPR133. (A) Schematic showing the extracellular N terminus, 7-transmembrane domain, and cytosolic C terminus of human GPR133. (B) GPR133 immunohistochemistry in a GBM specimen using (i) blocking peptide only, (ii) antibody only, (iii–v) antibody and different concentrations of blocking peptide. GPS, GPCR proteolysis site; GAIN, GPCR autoproteolysis-inducing domain.

IHC was performed on FFPE 4-µm-thick specimen sections, about 2–3 mm in diameter, using a mouse monoclonal anti-human GPR133 antibody (clone 8E3E8; IgG1ĸ isotype) that we previously described.^6^ This antibody was raised against peptide VNKGIYLKEEKGVTLLYYGRYNSSCISKPEQCGPEGVTFSFFWKTQGEQSRPIPSAYGGQVISNGFKVCSSGGRGSVELYTRDNSMTWEASFSPPGPYWTHVLFTWKSKEGLKVYVNGTLSTSDPSGKVSRDYGESNVNLVIGSEQDQAK within the pentraxin domain of the N terminus of GPR133 (Figure 1A).

The GPR133 antibody was optimized on GBM samples using chromogenic IHC performed on a Ventana Medical Systems Discovery XT instrument using Ventana’s reagents and detection kits unless otherwise noted. Slides were deparaffinized online and did not require antigen retrieval. Endogenous peroxidase activity was inactivated with 3% hydrogen peroxide for 4 min. The GPR133 antibody was prediluted to a concentration of 0.225 µg/mL in Tris-buffered saline (25 mM Tris, 0.15 mM NaCl, pH 7.2) with 1% bovine serum albumin and incubated overnight at 4°C. The antibody was subsequently applied to slides and incubated for 3 h at 37°C. The primary antibody was detected using an anti-mouse IgG/horseradish peroxidase multimer in an 8-min incubation. The complex was visualized with 3,3′-diaminobenzidine/H_2_O_2_ and enhanced with copper sulfate. Antibody validation consisted of pre-incubating the GPR133 antibody with the immunizing peptide 0.5 µg/mL (0.5×), 1 µg/mL (1×), and 2 µg/mL (2×). Weak labeling was observed at 0.5×, and no signal was detected at 1× or 2× (Figure 1B). A negative control consisted of applying antibody diluent or peptide alone to the samples in the absence of antibody. All slides were washed in distilled water, counterstained with hematoxylin, dehydrated, and mounted with permanent media.

### Quantification of IHC Signal

Quantification was performed by a neuropathologist (D.Z.), as previously described.^19,20^ Briefly, each slide was graded on a scale from 0 to ++++ based on the percentage of tumor cells that stained positive within the tissue: + (scored as 1), <1%; ++ (scored as 2), 1–10%; +++ (scored as 3), 10–50%; ++++ (scored as 4), >50%. For certain cases, the pathologist had to give 2 grades to a slide to account for tumors with heterogeneous GPR133 expression. In essence, 2 different representative areas were scored. For quantification purposes, the average of the 2 scores was used in such cases. For example, a +++/++++ grade was scored as 3.5, whereas a ++/++++ grade was given a 3.

To account for brain infiltration, we separately graded the infiltrating edge of tumors, when available, to characterize the expression pattern at the tumor periphery. Separately grading the infiltrating edge of each tumor also prevented the low tumor cell content and relatively lower staining at the tumor edge from skewing our results of staining within the bulk of the tumor.

### TCGA Data Analysi*s*

*GPR133 (ADGRD1*) mRNA expression data were obtained from the TCGA RNA-seq datasets for GBM and low-grade glioma (LGG).^8,10^ In addition, we collected data on *IDH1* and *IDH2* mutations, as well as 1p19q codeletion. Junction quantification data were obtained from the Broad Institute TCGA Genome Data Analysis Center (gdac.broadinstitute.org). Genomic coordinates of junctions were aligned to exons of all *GPR133* (*ADGRD1*) isoforms identified by RNA-seq.

### Statistics

Statistical comparisons were performed with non-parametric Kruskal–Wallis test followed by post hoc Dunn’s multiple comparisons and Mann–Whitney test. Statistical significance was set at *P* < .05. Dot plot graphs indicate the median, as well as an interquartile range with error bars. The numerical value depicted for each group in the graphs denotes the median value.

## Results

### Demographics

This study included a total of 67 gliomas and 6 non-neoplastic brain controls. Gliomas of all WHO grades were included: 6 grade I, 15 grade II, 5 grade III, and 41 grade IV. These included an assortment of glioma types (Supplementary Table 1):

Grade I: 5 juvenile pilocytic astrocytomas (JPA); 1 ganglioglioma;

Grade II: 1 pilomyxoid pilocytic astrocytoma; 1 pleomorphic xanthoastrocytoma; 3 *IDH* wild-type grade II diffuse astrocytomas; 3 grade II *IDH* mutant 1p19q codeleted oligodendrogliomas; 7 grade II *IDH* mutant 1p19q intact diffuse astrocytomas;

Grade III: 2 grade III *IDH* mutant anaplastic oligodendrogliomas; 3 grade III anaplastic astrocytomas, 2 of which were *IDH* mutant;

Grade IV: 34 grade IV *IDH* wild-type GBM; and 7 grade IV *IDH* mutant GBM. One of the *IDH* wild-type GBM specimens, located in the right thalamus, had a DNA methylation signature consistent with H3 K27M mutant glioma, even though the relevant immunohistochemical study was not performed. All tumors were cranial, except one cervical spinal cord pilocytic astrocytoma.

Thirty (44.8%) tumor samples were obtained from female patients, while 37 (55.2%) were obtained from males. The average patient age was 50.2 ± 2.7 years (median 57 years; range 4–86 years). Of the 67 patients, only 5 were pediatric (age <18 years). Of these pediatric patients, 4 had grade I JPA and 1 had a cerebellar grade II *IDH* wild-type astrocytoma.

### GPR133 Is Not Expressed in Non-neoplastic Brain Tissue

**Figure 2.**
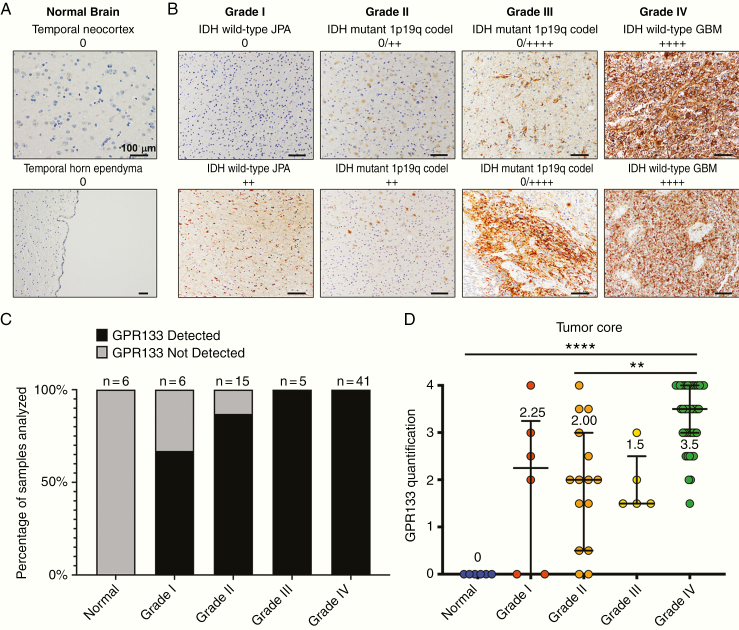
Non-neoplastic brain tissue lacks GPR133 expression, whereas gliomas express GPR133 at higher levels with increasing WHO grade. (A) GPR133 immunohistochemistry in temporal neocortex and temporal horn ependyma in representative control specimens. Scale bar, 100 μm. (B) GPR133 antibody stains in gliomas grade I–IV. Scale bar, 100 μm. (C) Proportion of samples with GPR133 expression by WHO grade. (D) Level of GPR133 expression by grade (*P* < .0001, Kruskal–Wallis test; post hoc Dunn’s **P < .003; ****P < .0001). codel: codeleted.

None (0/6) of the non-neoplastic brain samples derived from autopsy or a temporal lobectomy for resection of cortical dysplasia causing seizures showed any immunostaining for GPR133 (Figure 2A). Negative staining was obtained not only in cortical tissue and subcortical white matter, but also in the subventricular zone, where neural progenitors, which are thought to give rise to gliomas, reside^15^ (Figure 2A). These data are consistent with available single-cell RNA-seq (SMART-seq) data from human brain cells in the Allen Brain Map (portal.brain-map.org) (Supplementary Figure 1A) and bulk RNA-seq of purified populations of human brain cell types in the Brain RNA-seq (brainrnaseq.org) database^21^ (Supplementary Figure 1B). Briefly, the transcriptome information indicates no *GPR133* mRNA in human neurons, astrocytes, and oligodendrocytes. Low amounts of *GPR133* mRNA may be found only in microglia, endothelial cells, and pericytes among the resident brain cell types.

### The Anti-GPR133 Antibody Recognizes the Major GPR133 Splice Variants in Glioma

We examined the expression of *GPR133* (*ADGRD1*) mRNA in the RNA-seq dataset of the TCGA for diffuse glioma.^8,10^ As shown in Supplementary Figure 2A, *IDH* wild-type gliomas have significantly higher amounts of *GPR133* transcript than *IDH* mutant non-codeleted astrocytic tumors, consistent with our findings at the protein level.

The *GPR133* (*ADGRD1*) gene contains 26 exons and GenBank predicts several splice variants (Supplementary Figure 2B). We analyzed the GBM and LGG RNA-seq dataset in the TCGA^8^ to identify the predominant splice variants. Exon junction analysis indicated that the primary isoforms are comprised of either 25 or 26 exons (uc001uit.4 and uc010tbm.2) (Supplementary Figure 2C). The alternative splicing in these isoforms involves the inclusion of an additional exon (“3a”), encoding part of the N terminus, in transcript uc010tbm.2. These 2 isoforms encode 874 and 906 aa proteins (Q6QNK2-1 and Q6QNK2-4, respectively, on Uniprot), with a large extracellular N terminus, a 7-transmembrane region, and a cytosolic C terminus. The mouse antibody used in this study was raised against a sequence within the pentraxin domain of the N terminus, which is encoded in both of these long splice variants. This indicates that our antibody is able to detect the predominant *GPR133* splice variants in glioma. The short variants, which are less abundant in tumors based on our exon junction analysis, are not predicted to be recognized by the antibody used in this study.

### GPR133 Expression in Gliomas

**Figure 3.**
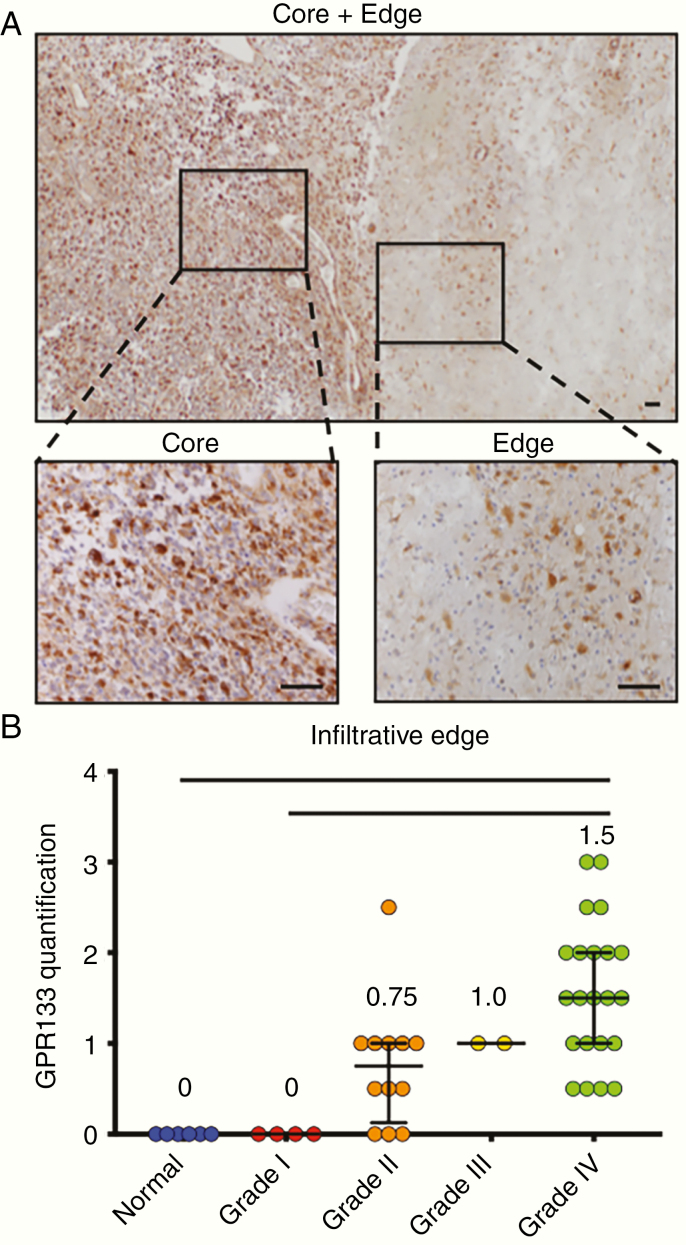
GPR133 is detected in both the core and infiltrative edge of gliomas, as well as in areas of pseudopalisading necrosis. (A) GPR133 immunohistochemistry in *IDH* wild-type GBM tissue at the core (left zoom) and infiltrative edge (right zoom). The tumor core was graded as +++, and the tumor edge was graded +/++. Scale bar, 100 μm. (B) GPR133 quantification at the infiltrative edge by grade (*P* < .0001, Kruskal–Wallis test; post hoc Dunn’s *P < .01; **P < .001). Scale bar, 100 μm.

Using our antibody and chromogenic IHC, we found that the prevalence of expression of GPR133 within the tumor bulk increased with WHO tumor grade (Figure 2B–D). All grade III and IV tumors showed GPR133 immunoreactivity, with grade IV having the highest levels. In contrast, 33% (2/6) grade I and 13% (2/15) grade II had no GPR133 detected. GPR133 expression was seen both within the tumor bulk and along the infiltrative edge in grades II–IV (Figure 3A and B).

We previously showed that *GPR133* transcription is upregulated in hypoxia by transcription factor HIF1α.^6,7^ We therefore tested whether GPR133 expression is enriched in areas of pseudopalisading necrosis, which are thought to be the most hypoxic territories within GBM tumors. Indeed, while GPR133 is diffusely expressed in GBM tumors, including within the tumor bulk and infiltrating edges, we qualitatively identified increased expression in these pseudopalisading cells surrounding necrotic cores (Supplementary Figure 3). This confirmed our previous observations.^6,7^

### GPR133 Expression in Molecular Subtypes of Glioma

**Figure 4.**
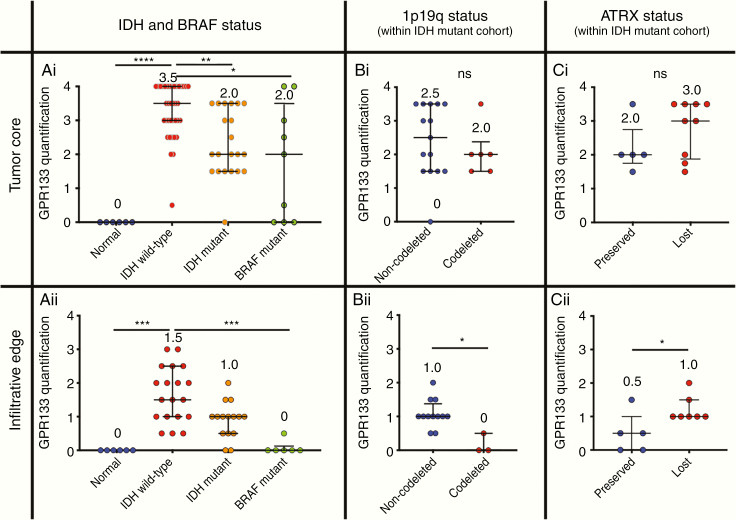
Influence of IDH and BRAF mutations on GPR133 expression. (Ai) GPR133 expression, as assessed by immunohistochemistry, is higher in the core of *IDH* wild-type gliomas compared to *IDH* mutant and *BRAF* mutant gliomas (*P* < .0001, Kruskal–Wallis test; post hoc Dunn’s *P < .05; **P < .01; ****P < .0001). (Aii) Similar comparisons in the infiltrative edge of such tumors (*P* = <.0001, Kruskal–Wallis test; post hoc Dunn’s ***P < .0005). (Bi) There is no difference in GPR133 expression within the core of 1p19q codeleted versus non-codeleted *IDH* mutant gliomas (Mann–Whitney test; ns, *P* > .05). (Bii) The infiltrative edge of non-codeleted *IDH* mutant gliomas shows higher levels of GPR133 expression (Mann–Whitney test; *P < .05). (Ci) GPR133 expression is equivalent within the core of *IDH* mutant gliomas with preserved or lost *ATRX* (Mann–Whitney test; ns, *P* > .05). (Cii) The infiltrative edge of *ATRX* loss *IDH* mutant gliomas shows higher levels of GPR133 expression (Mann–Whitney test; *P < .05).

We then analyzed the expression of GPR133 across glioma molecular subtypes, as defined by the *IDH1/2* mutations, 1p19q codeletion, and *BRAF* status. Of note, in this study, the *BRAF* mutant group includes tumors either directly confirmed for the *BRAF* V600E mutation by IHC or NGS, or tumor histologies where *BRAF* mutations are frequent (eg, JPA). Expression of GPR133 was significantly higher in the core (bulk) of *IDH* wild-type gliomas, when compared to *IDH* mutant (including both 1p19q codeleted and non-codeleted subgroups) and *BRAF* mutant tumors (Figure 4Ai). The differences between *IDH* wild-type and *BRAF* mutant tumors in expression were also seen in the infiltrative edge of tumors (Figure 4Aii).

When *IDH* mutant tumors were further divided into 1p19q non-codeleted (astrocytomas) and codeleted (oligodendrogliomas) tumors, GPR133 expression was equivalent within the tumor core (Figure 4Bi), but elevated in the infiltrative edge of non-codeleted specimens (Figure 4Bii). Similarly, when we assessed GPR133 expression as a function of *ATRX* status, there was no difference within the core of *IDH* mutant gliomas with preserved (oligodendrogliomas) or lost (astrocytomas) *ATRX* (Figure 4Ci). However, GPR133 expression was higher in the infiltrative edge of gliomas with *ATRX* loss (Figure 4Cii).

**Figure 5.**
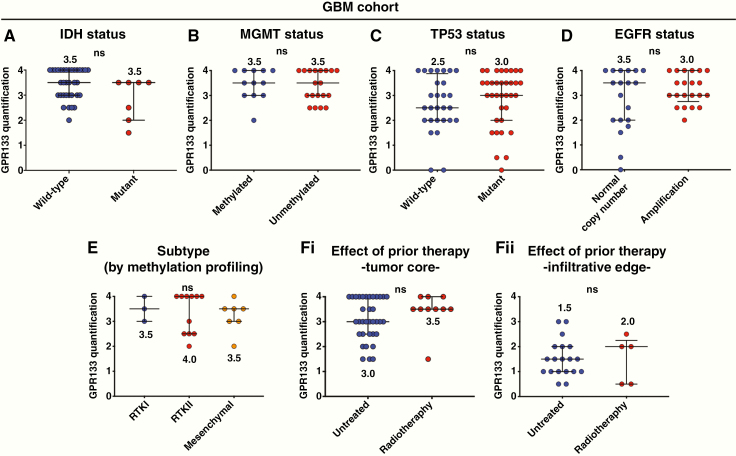
Influence of molecular markers and prior therapy on GPR133 expression within the GBM cohort. (A–D) *IDH* mutation, *MGMT* promoter methylation, TP53 immunohistochemical signal, and *EGFR* amplification had no effect on GPR133 expression within the core of GBM tumors (Mann–Whitney test; ns, *P* > .05). (E) No difference in GPR133 tumor core expression was found among RTK I, RTK II, and mesenchymal subtypes of gliomas (Kruskal–Wallis test; ns, *P* = .9876). (Fi and ii) There was no difference between newly diagnosed and previously treated recurrent GBM within the tumor core (i) or infiltrative edge (ii) (Mann–Whitney test; ns, *P* > .05).

Within the GBM cohort, there was no significant difference in GPR133 expression between *IDH* wild-type and mutant grade IV (GBM) tumors (Figure 5A). The *MGMT*, TP53, and *EGFR* status did not influence GPR133 expression (Figure 5B–D). Furthermore, we examined the influence of molecular subtype as defined by profiling with DNA methylation arrays.^16,17^ GPR133 expression did not differ among the RTK I, RTK II, and mesenchymal subtypes (Figure 5E). We did not identify a significant effect of prior treatment (radiotherapy and/or chemotherapy)^22^ on GPR133 expression within the tumor core or infiltrative edge (Figure 5Fi, ii).

## Discussion

This is the first study describing the expression profile of GPR133 throughout the various histopathologic and molecular subtypes of the glioma family. Our work suggests that GPR133 is de novo expressed in glioma, given it is essentially absent in normal brain. Available RNA-seq data from human brain cells support our IHC finding that GPR133 is not expressed in neurons, astrocytes, or oligodendrocytes and indicate only low levels of *GPR133* transcript in microglia and endocytes/pericytes. In gliomas, the expression of GPR133 correlates with increasing WHO grade and, therefore, anaplasia. Indeed, levels of GPR133 are highest in GBM, where it is expressed in all tumors interrogated. Furthermore, both the tumor bulk and brain-infiltrating tumor edge, major treatment targets that cannot be addressed surgically, express GPR133. The fact that GPR133 is ubiquitously found in high-grade glioma, whether *IDH* wild-type or mutant, but is absent in non-neoplastic brain, supports our position that it may be exploited therapeutically.

Our work does not clearly define the nature of the cells that express GPR133 either within the tumor bulk or the infiltrative edge. The fact that GPR133 is expressed in patient-derived GBM cultures suggests that it is present in tumor cells. However, it is not clear whether tumor-associated immune cells, microglia, vascular lineages, or reactive astrocytes may also express GPR133. A future project of ours will be to define the cell lineages in which GPR133 is present, both within the tumor bulk and also along the brain-infiltrative edge.

Our previous work suggested that GPR133 expression is enriched in CD133-positive stem-like GBM cells.^6^ While the CD133-positive fraction is usually a minority of the total tumor cell population, the implication is that GPR133 is still expressed in CD133-negative cells, so that, overall, it is expressed in the majority of tumor cells. This is supported by our current immunohistochemical observations that suggest that GPR133 is expressed in over 50% of cells within the tumor bulk in the majority of GBM specimens.

Within GBM, GPR133 expression is not related to *MGMT* methylation, *EGFR* amplification, TP53 status, molecular subtype, or even IDH status, indicating potential generalizability of its use therapeutically and even diagnostically. Potential diagnostic applications of GPR133 include routine immunohistochemical analysis of glioma specimens and even intraoperative visualization of GPR133 via fluorophore-conjugated antibodies. This latter possibility is based on the lack of GPR133 detection in non-neoplastic brain, suggesting a potential use as a binary diagnostic tool in glioma surgery. The fact that GPR133 expression is not altered by prior chemoradiotherapy suggests that it may be a promising target in both the newly diagnosed and recurrent settings in GBM. Within the whole spectrum of tumors in the glioma family, however, we do observe trends toward higher GPR133 expression in *IDH* wild-type glioma versus *IDH* mutant astrocytic and oligodendroglial tumors. This trend is consistent with TCGA RNA-seq data.

Splice variant analysis from TCGA data indicates that 2 long isoforms of *GPR133* are the predominant species in glioma (encoding 874 and 906 aa proteins, respectively). Both of these isoforms encode a long N terminus, the 7-transmembrane region, and the cytosolic C terminus. In addition, both transcript variants are predicted to be recognized by our antibody. It is unclear what the function of shorter splice variants may be. A recent study suggested that shorter transcripts that do not encode the long N terminus are generated by an alternative in-gene promoter and may not be able to signal through G proteins.^23^ Future research will have to be dedicated to the whole gamut of *GPR133* transcript variants.

One limitation of our work is the modest size of our specimen cohort, particularly for WHO grades I–III. This size limitation impacts the statistical power or our analysis, particularly when small groups are being compared. Nonetheless, we feel that this immunohistochemical survey for GPR133 makes the point that it is widely expressed in the glioma family but is not present in non-neoplastic brain tissue.

In summary, the current study provides additional evidence for GPR133 as a novel treatment option in glioma. Several therapeutic approaches may be appropriate, ranging from small molecule inhibitors to biologics modulating the GPR133 function. However, even without considering function-modulating therapies, the localization of GPR133 on the cell surface potentially allows for the development of targeted immuno-oncologic therapies, such as antibody–drug complexes, bi-specific T-cell engagers, or chimeric antigen receptor T cells. Trials of immune checkpoint inhibitors, which are agnostic to specific antigens, have so far shown limited efficacy in GBM, with very few exceptions.^24^ We therefore postulate that GPR133 may present an opportunity for targeted immune therapies. One of the limitations in considering such approaches is the fact that GPR133 is expressed in several extracranial tissues,^6^ raising the possibility of systemic toxicity. Future research will be necessary to assess this concern and, if needed, circumvent it via intrathecal administration of therapeutics.

## Funding

This study was supported by National Institutes of Health/National Institute of Neurological Disorders and Stroke (R01 NS102665) and NYSTEM (NY State Stem Cell Science) IIRP (C32595GG to D.G.P.). D.G.P. was also supported by National Institutes of Health/National Institute of Allergy and Infectious Diseases (R21 AI130618) and DFG (German Research Foundation) (FOR2149). J.D.F. was supported by a NYSTEM Training Grant. L.C. was supported by National Institutes of Health/National Cancer Institute (P33CA016087), National Institutes of Health/Office of Research Infrastructure Programs (S10OD01058), and NIH/ORIP (S10OD018338). G.S. was supported by a DFG postdoctoral fellowship (STE 2843/1-1). C.Y.P. was supported by a Leukemia and Lymphoma Society Scholar Award. I.L. and T.S. were supported by DFG FOR2149 (project numbers 266022790 P4 to T.S. and P5 to I.L.) and CRC1052 (project number 209933838 B6 to I.L. and T.S.). The methylation profiling was in part supported by a grant from the Friedberg Charitable Foundation to M.S.

*Conflict of interest statement*. D.G.P., D.Z., NYU Grossman School of Medicine, and Heptares have filed a patent application titled “Method for treating high grade glioma” on the use of GPR133 as a treatment target in glioma. D.G.P. has received consultant fees from Tocagen, Synaptive Medical, Monteris, and Robeaute. A.S.C. is currently an employee of Neon Therapeutics. G.W. and M.B. hold shares and/or share options in Sosei Heptares.

## Authorship Statement

J.D.F., M.K., S.K., and J.S.: performed experiments, analyzed data, and participated in manuscript preparation. L.C., D.B., D.G., N.R.B., and G.S.: performed experiments and edited manuscript. A.S.C., S.C.K., R.J., E.S., C.Y.P., D.F., M.S., I.L., T.S., G.W., R.N., M.B., D.J.M., J.K.D., X.H., and N.S.: contributed to experimental design and edited manuscript. D.Z.: experimental design, analyzed data, and participated in manuscript preparation. D.G.P.: conceived the study, experimental design, analyzed data, and manuscript preparation.

## Supplementary Material

vdaa053_suppl_Supplementary_Figure_1

vdaa053_suppl_Supplementary_Figure_2

vdaa053_suppl_Supplementary_Figure_3

vdaa053_suppl_Supplementary_Materials

vdaa053_suppl_Supplementary_Table_1
