# Supplementary figures and images for: Expression profiling of the adhesion G protein-coupled receptor GPR133 (ADGRD1) in glioma subtypes
Source: Neurooncol Adv. 2020 Apr 28;2(1):vdaa053. doi: 10.1093/noajnl/vdaa053 (PMC7262742; doi:10.1093/noajnl/vdaa053)

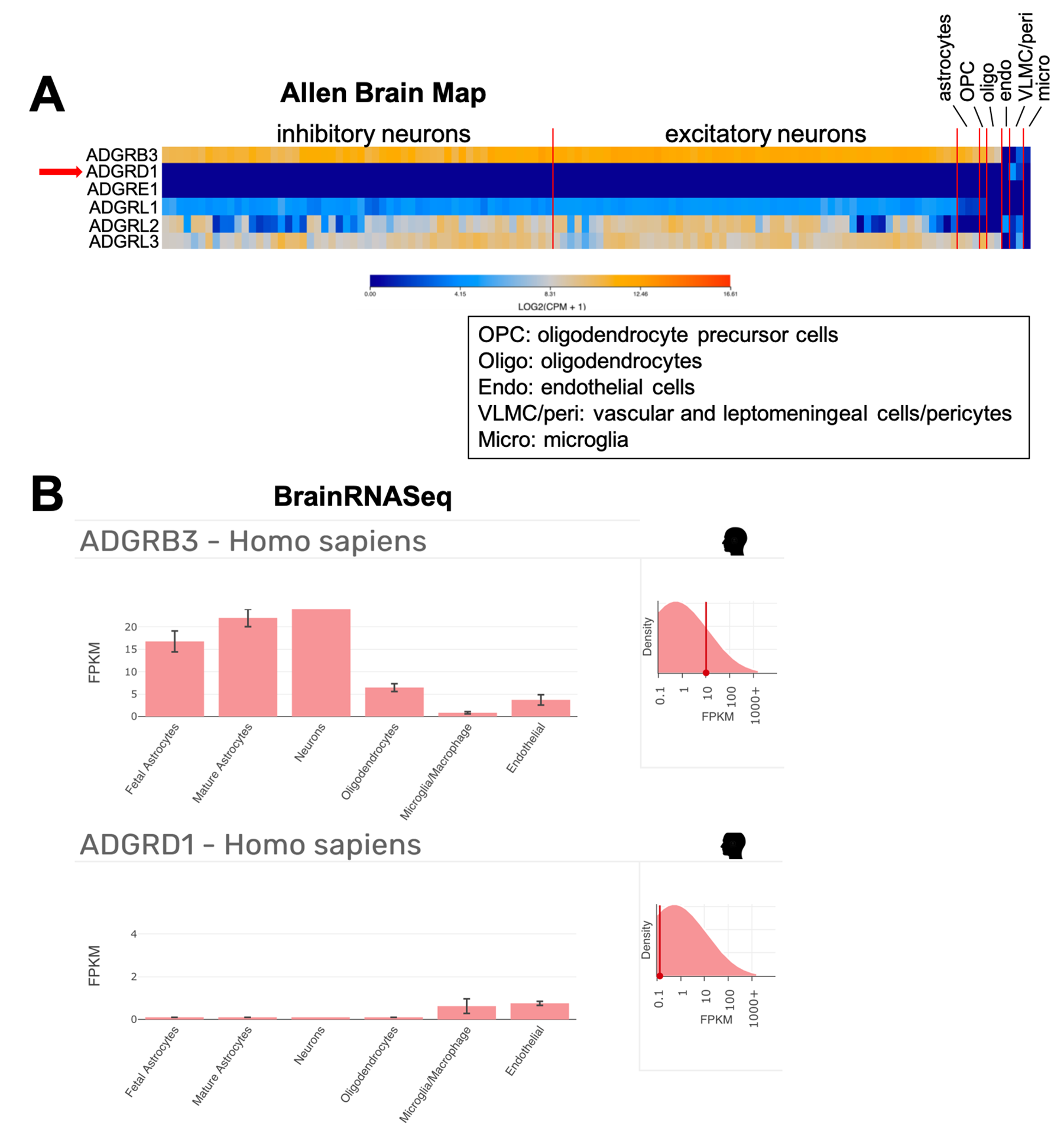

Supplement: vdaa053_suppl_Supplementary_Figure_1 [file vdaa053_suppl_supplementary_figure_1.png]

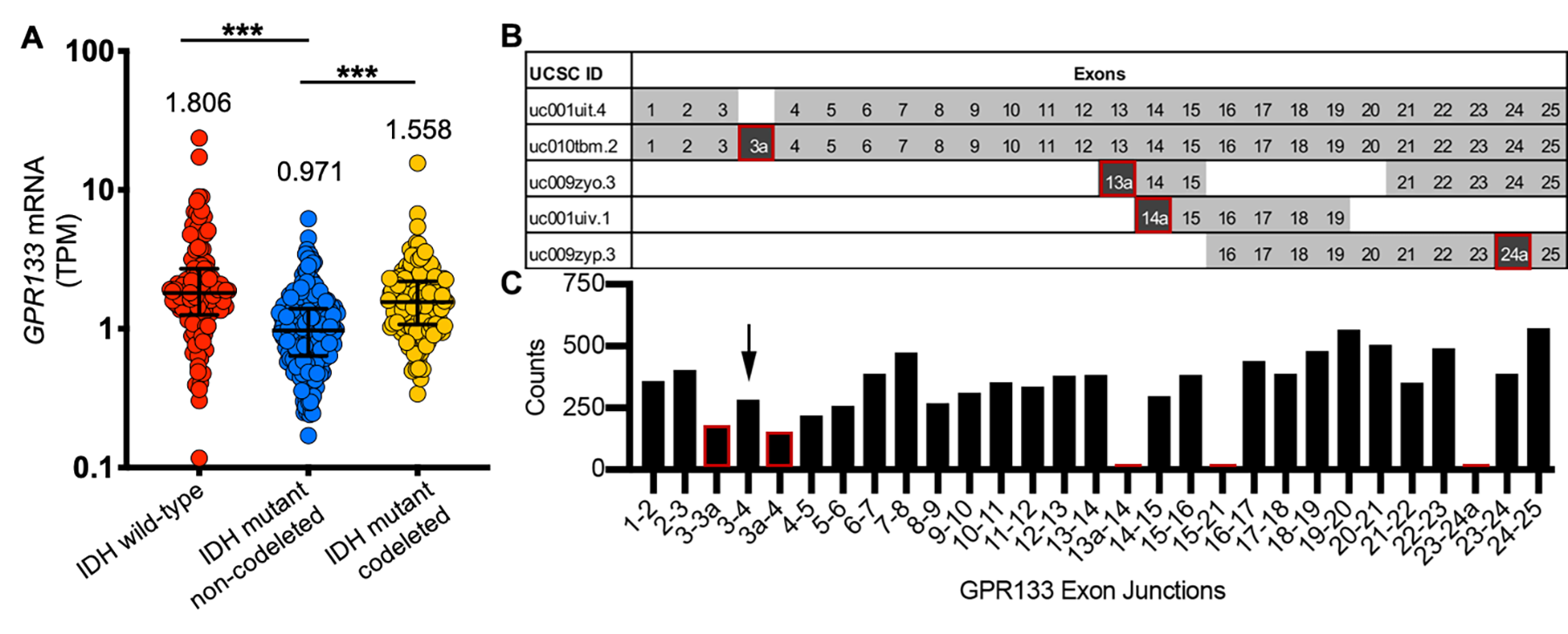

Supplement: vdaa053_suppl_Supplementary_Figure_2 [file vdaa053_suppl_supplementary_figure_2.png]

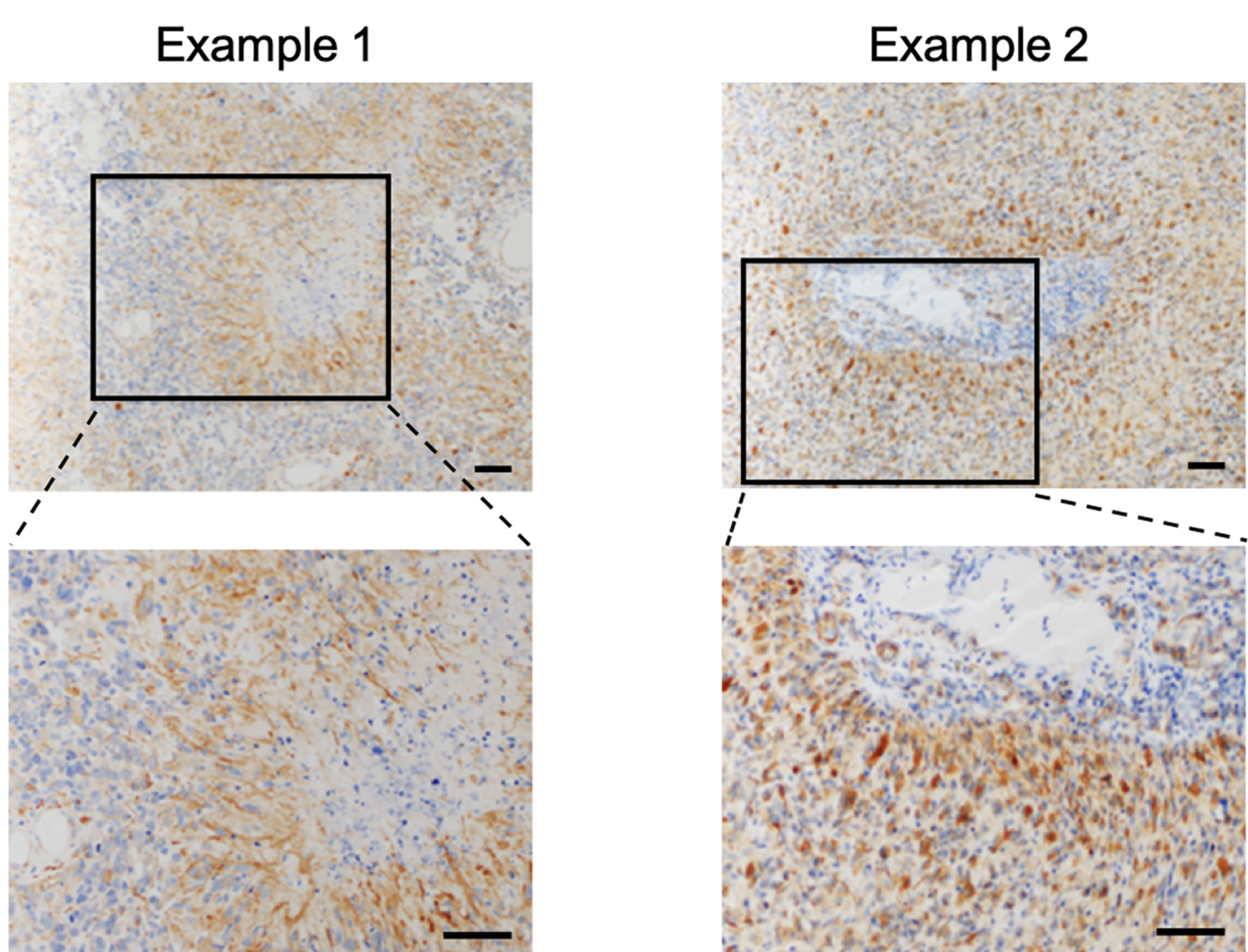

Supplement: vdaa053_suppl_Supplementary_Figure_3 [file vdaa053_suppl_supplementary_figure_3.png]
