# Supplementary material for: Expression profiling of the adhesion G protein-coupled receptor GPR133 (ADGRD1) in glioma subtypes
Source: Neurooncol Adv. 2020 Apr 28;2(1):vdaa053. doi: 10.1093/noajnl/vdaa053 (PMC7262742; doi:10.1093/noajnl/vdaa053)
Supplement: vdaa053_suppl_Supplementary_Materials [file vdaa053_suppl_supplementary_materials.docx]

**SUPPLEMENTARY MATERIAL**

**Supplementary Table**

**Supplementary Table 1. List of specimens with demographic/clinical information, diagnostic pathology, relevant markers and GPR133 expression levels.**

+, -: positive and negative diagnostic test

Term: nonsense mutation

**Supplementary Figure Legends**

**Supplementary Figure 1. *GPR133* mRNA is not found in neurons, astrocytes or oligodendrocytes in the human brain. A.** Single cell RNA-seq in the Allen Brain Map indicates no expression of *GPR133* (*ADGRD1*) mRNA in human neurons, astrocytes, oligodendrocyte precursor cells (OPC), and oligodendrocytes. Limited expression may be found in pericytes (VLMC/peri). The expression profile of other adhesion GPCRs is shown for comparison. **B**. RNA-seq data in the brainrnaseq database indicates only limited expression of *GPR133* (*ADGRD1*) mRNA in microglia and endothelial cells in the human brain. There is no *GPR133* expression in neurons, astrocytes, and oligodendrocytes. The expression profile of the adhesion GPCR *ADGRB3* mRNA is shown for comparison.

**Supplementary Figure 2. Analysis of TCGA RNA-seq data.** **A.** Among the TCGA lower grade glioma cohort (n=523), *GPR133* mRNA is higher in *IDH1* wild-type compared to *IDH1* mutant non-codeleted gliomas (P<0.0001, Kruskal-Wallis test; post hoc Dunn’s ***, P<0.0001). **B.** Exon map for five *GPR133* mRNA splice variants in the UC Santa Cruz gene database shows two long and three shorter transcript variants. Exons unique to specific splice variants are boxed in red. **C.** Exon junction analysis from the Broad GDAC Firehose of the TCGA GBM cohort shows that the two predominant *GPR133* mRNA species, uc001uit.4 and uc010tbm.2, contain 25 and 26 exons (black arrow points to junction between exons 3 and 4 in the 25 exon variant, uc001uit.4; an additional exon, 3a, is included in variant uc010tbm.2) and include the large N terminus, which is the target of the antibody used in this study. The three shorter transcript variants appear much less abundant and are not predicted to be recognized by the antibody used in this study.

**Supplementary Figure 3. Strong GPR133 expression in areas of pseudopalisading necrosis.** GPR133 antibody stain of pseudopalisading necrosis in two *IDH* wild-type GBM specimens. Scale bar, 100 μm.
