# Supplementary material for: Expression profiling of the adhesion G protein-coupled receptor GPR133 (ADGRD1) in glioma subtypes
Source: Neurooncol Adv. 2020 Apr 28;2(1):vdaa053. doi: 10.1093/noajnl/vdaa053 (PMC7262742; doi:10.1093/noajnl/vdaa053)
Supplement: vdaa053_suppl_Supplementary_Table_1 [file vdaa053_suppl_supplementary_table_1.docx]

| **Case number** | **Age** | **Sex** | **Location** | **Diagnosis** | **Newly diagnosed vs. recurrent** | **WHO grade** | **GPR133 score** | | **Immunohistochemistry markers** | | | | **Molecular markers** | | | **DNA methylation profile** | | **NGS** |
| --- | --- | --- | --- | --- | --- | --- | --- | --- | --- | --- | --- | --- | --- | --- | --- | --- | --- | --- |
|  |  |  |  |  |  |  | **Tumor core** | **Infiltrative edge** | **IDH1 R132H** | **TP53** | **ATRX** | **BRAF V600E** | **EGFR** | **1P19Q codeletion** | **MGMT** | **DNA methylation classifier** | **classifier score** |  |
| **Tumor cases** | | | | | | | | | | | | | | | | | | |
| 1 | 12 | F | Left Brainstem | Juvenile Pilocytic Astrocytoma | newly diagnosed | I | 2 | N/A | WT | - | not done | - | not done | not done | not done | pilocytic astrocytoma | 0.98 | APC E1317Q |
| 2 | 6 | M | Cerebellum | Juvenile Pilocytic Astrocytoma | newly diagnosed | I | 0 | 0 | WT | - | not done | - | not done | not done | not done | control tissue cerebellar hemisphere | 0.99 | None |
| 3 | 4 | F | Hypothalamus | Juvenile Pilocytic Astrocytoma | newly diagnosed | I | 4 | 0 | WT | - | preserved | - | not done | not done | not done | not done | N/A | None |
| 4 | 4 | F | Not Specified | Pilocytic Astrocytoma | newly diagnosed | I | 2.5 | N/A | WT | - | not done | - | not done | not done | not done | not done | N/A | not done |
| 5 | 18 | M | Cervical cord | Glioma w/ Pilocytic Features | newly diagnosed | I | 0 | 0 | WT | not done | not done | not done | not done | not done | not done | not done | N/A | not done |
| 6 | 36 | F | Left Temporal | Ganglioglioma | newly diagnosed | I | 3 | 0 | WT | - | preserved | + | not done | not done | not done | control tissue hemispheric cortex | 0.68 | BRAF V600E |
| 7 | 16 | M | Right 3rd Ventricle | Pilomyxoid Pilocytic Astrocytoma | newly diagnosed | II | 0 | 0 | WT | - | preserved | - | not done | not done | not done | Pilocytic astrocytoma | 0.68 | not done |
| 8 | 17 | F | Left Temporal | Pleomorphic Xanthoastrocytoma | newly diagnosed | II | 4 | N/A | WT |  | preserved | + | not done | not done | not done | pleomorphic xanthoastrocytoma | 0.99 | BRAF V600E |
| 9 | 5 | M | Left Cerebellum | Infiltrating Fibrillary Astrocytoma | newly diagnosed | II | 2.5 | 2.5 | WT | - | preserved | - | not done | not done | not done | NO MATCH | <0.3 | not done |
| 10 | 46 | F | Right Temporal | Diffuse Astrocytoma | newly diagnosed | II | 0.5 | 0.5 | WT | + | not done | - | - | - | Methylated | low grade glioma ganglioglioma | 0.56 | BRAF V600E |
| 11 | 50 | F | Right, Unspecified | Oligoastrocytoma | newly diagnosed | II | 0.5 | 0.5 | WT | some + | preserved | - | not done | - | Methylated | not done | N/A | not done |
| 12 | 61 | F | Left Frontal | Oligodendroglioma | newly diagnosed | II | 2 | 0 | MUT | - | not done | - | not done | + | not done | NO MATCH | <0.3 | IDH1 R132H |
| 13 | 21 | F | Right, Unspecified | Oligodendroglioma | newly diagnosed | II | 2 | 0 | MUT | - | preserved | - | not done | + | not done | Glioma IDH mutant | 0.99 | IDH1 R132H |
| 14 | 48 | M | Left Frontal | Oligodendrioglioma | newly diagnosed | II | 2 | 0.5 | MUT | - | not done | - | not done | + | not done | not done | N/A | IDH1 R132H |
| 15 | 26 | F | Left Temporal | Diffuse Astrocytoma | newly diagnosed | II | 0 | 1 | MUT | + | not done | - | - | - | Unmethylated | Glioma IDH mutant | 0.99 | not done |
| 16 | 33 | F | Left Frontal | Diffuse Astrocytoma | unknown | II | 3.5 | N/A | MUT | + | lost | not done | not done | - | not done | Glioma IDH mutant | 0.99 | Inadequate Tissue |
| 17 | 35 | M | Right Temporal | Astrocytoma | newly diagnosed | II | 1.5 | 1.5 | MUT | + | lost | - | - | - | Unmethylated | Glioma IDH mutant | 0.99 | IDH1 R132H; PIK3CA E542K |
| 18 | 31 | F | Left Frontal | Astrocytoma | newly diagnosed | II | 3.5 | 1 | MUT | + | lost | not done | not done | - | not done | Glioma IDH mutant | 0.99 | IDH1 R132H; NOTCH1 L2457V |
| 19 | 51 | F | Right Frontal | Infiltrating astrocytoma | newly diagnosed | II | 2 | 1 | MUT | + | not done | - | - | - | Unmethylated | Glioma IDH mutant | 0.99 | not done |
| 20 | 67 | F | Right Frontal | Gemistocytic Astrocytoma | newly diagnosed | II | 3 | 1 | MUT | + | lost | - | not done | not done | not done | NO MATCH | <0.3 | IDH1 R132H; PIK3CA I1019T; TP53 G245S |
| 21 | 37 | F | Left Frontal | Diffuse Astrocytoma with focal anaplasia | unknown | II | 1.5 | N/A | MUT | + | lost | not done | not done | - | not done | Glioma IDH mutant | 0.99 | IDH1 R132H; TP53 R273C |
| 22 | 60 | M | Right, Unspecified | Anaplastic Astrocytoma | newly diagnosed | III | 2 | 1 | WT | Focal + | lost | not done | + | not done | not done | Glioblastoma IDH wildtype | 0.86 | Failed |
| 23 | 26 | M | Right Temporal | Anaplastic Astrocytoma | newly diagnosed | III | 3 | 1 | MUT | + | lost | - | not done | not done | not done | NO MATCH | <0.3 | not done |
| 24 | 62 | F | Right Temporal | Anaplastic Oligoastrocytoma | newly diagnosed | III | 1.5 | N/A | MUT | + | not done | not done | not done | - | not done | not done | N/A | not done |
| 25 | 50 | M | Right Frontal | Anaplastic Oligodendroglioma | newly diagnosed | III | 1.5 | N/A | MUT | Rare + | not done | - | not done | + | not done | NO MATCH | <0.3 | not done |
| 26 | 56 | M | Right Frontal | Anaplastic Oligodendroglioma | recurrent | III | 1.5 | N/A | MUT | - | not done | - | not done | + | not done | Glioma IDH mutant | 0.99 | IDH1 R132S; APC E1317Q |
| 27 | 62 | F | Right Temporal | Glioblastoma | recurrent | IV | 4 | 2.5 | WT | + | not done | not done | not done | not done | Methylated | Glioblastoma IDH wildtype | 0.99 | not done |
| 28 | 67 | F | Right Temporal | Glioblastoma | newly diagnosed | IV | 4 | 2.5 | WT | + | not done | not done | + | - | Unmethylated | not done | N/A | not done |
| 29 | 77 | M | Left Temporal | Glioblastoma | newly diagnosed | IV | 3 | N/A | WT | Rare + | not done | - | + | not done | Unmethylated | Glioblastoma IDH wildtype | 0.99 | not done |
| 30 | 14 | M | Right Thalamus | Glioblastoma | newly diagnosed | IV | 4 | 2 | WT | + | not done | - | not done | - | not done | diffuse midline glioma H3 K27M mutant | 0.99 | not done |
| 31 | 86 | M | Right, Unspecified | Glioblastoma | newly diagnosed | IV | 3 | N/A | WT | + | not done | - | not done | + | Methylated | Glioblastoma IDH wildtype | 0.99 | not done |
| 32 | 52 | F | Left, Unsspecified | Glioblastoma | newly diagnosed | IV | 4 | N/A | WT | - | not done | - | + | not done | not done | Glioblastoma IDH wildtype | 0.98 | not done |
| 33 | 73 | F | Right Frontal | Glioblastoma | newly diagnosed | IV | 4 | N/A | WT | - | not done | - | - | not done | Methylated | Glioblastoma IDH wildtype | 0.99 | None |
| 34 | 56 | M | Left Frontal | Glioblastoma | newly diagnosed | IV | 3.5 | 1.5 | WT | Rare + | preserved | - | - | not done | not done | Glioblastoma IDH wildtype | 0.99 | not done |
| 35 | 63 | F | Right Parietal | Glioblastoma | newly diagnosed | IV | 4 | 3 | WT | Scattered + | preserved | - | + | not done | Unmethylated | Glioblastoma IDH wildtype | 0.99 | None |
| 36 | 39 | M | Left Brain | Glioblastoma | recurrent | IV | 3.5 | N/A | WT | Variable + | preserved | - | not done | not done | Unmethylated | Glioblastoma IDH wildtype | 0.99 | None |
| 37 | 76 | M | Left Brain | Glioblastoma | newly diagnosed | IV | 2.5 | N/A | WT | Scattered + | preserved | - | - | - | Unmethylated | Glioblastoma IDH wildtype | 0.99 | APC I1307K. |
| 38 | 61 | F | Left Parieto-occipital | Glioblastoma | newly diagnosed | IV | 4 | N/A | WT | + | not done | not done | not done | - | not done | Glioblastoma IDH wildtype | 0.88 | not done |
| 39 | 48 | F | Left Temporal | Glioblastoma | unknown | IV | 3.5 | N/A | WT | - | not done | not done | not done | - | Methylated | not done | N/A | not done |
| 40 | 66 | M | Right Parietal | Glioblastoma | newly diagnosed | IV | 3 | 1.5 | WT | + | preserved | - | + | not done | Unmethylated | not done | N/A | RB1 R358Term; TP53 C242S |
| 41 | 79 | M | Left Temporal | Glioblastoma | newly diagnosed | IV | 2 | 1 | WT | - | preserved | - | - | not done | Methylated | Glioblastoma IDH wildtype | 0.99 | PTEN E291TERM; JAK3 V718L |
| 42 | 59 | M | Left Temporal | Glioblastoma | newly diagnosed | IV | 2.5 | 1 | WT | - | preserved | - | + | not done | Unmethylated | Glioblastoma IDH wildtype | 0.93 | PTEN W111C |
| 43 | 60 | M | Right Parietal | Glioblastoma | newly diagnosed | IV | 4 | N/A | WT | - | preserved | - | - | not done | Unmethylated | Plexus Tumor | 0.33 | None |
| 44 | 66 | M | Left Frontal | Glioblastoma | recurrent | IV | 3.5 | N/A | WT | + | preserved | - | - | not done | Unmethylated | control tissue inflammatory | 0.99 | None |
| 45 | 30 | M | Right Temporal | Glioblastoma | newly diagnosed | IV | 2.5 | 2 | WT | - | preserved | - | + | not done | Unmethylated | Glioblastoma IDH wildtype | 0.99 | ATM F858L |
| 46 | 76 | F | Right Occipital | Glioblastoma | newly diagnosed | IV | 4 | 2 | WT | - | preserved | - | - | - | Unmethylated | Glioblastoma IDH wildtype | 0.45 | PTEN R15Term; TP53 R196Term |
| 47 | 71 | M | Left, Unspecified | Glioblastoma | newly diagnosed | IV | 2.5 | N/A | WT | - | preserved | - | + | - | Unmethylated | Glioblastoma IDH wildtype | 0.99 | EGFR Q105H; EGFR R108K |
| 48 | 65 | M | Right Frontal | Glioblastoma | newly diagnosed | IV | 3 | N/A | WT | - | preserved | - | + | - | Unmethylated | control tissue inflammatory tumor microenvironment | 0.99 | EGFR G719A |
| 49 | 71 | M | Left Frontal | Glioblastoma | newly diagnosed | IV | 3.5 | N/A | WT | + | preserved | - | - | - | Methylated | Glioblastoma IDH wildtype | 0.99 | EGFR A289V |
| 50 | 54 | M | Left Temporal | Glioblastoma | newly diagnosed | IV | 4 | N/A | WT | - | preserved | - | + | not done | Unmethylated | Glioblastoma IDH wildtype | 0.99 | None |
| 51 | 86 | M | Right Temporal | Glioblastoma | recurrent | IV | 3.5 | 0.5 | WT | + | preserved | - | - | - | Methylated | Glioblastoma IDH wildtype | 0.99 | not done |
| 52 | 61 | M | Left Temporo-occipital | Glioblastoma | newly diagnosed | IV | 3 | 0.5 | WT | - | preserved | - | + | - | unmethylated | Glioblastoma, IDH wildtype | 0.99 | APC P865S |
| 53 | 54 | M | Right Thalamus | Glioblastoma | newly diagnosed | IV | 4 | N/A | WT | - | preserved | - | + | not done | Methylated | Glioblastoma IDH wildtype | 0.88 | not done |
| 54 | 66 | F | Right Frontal | Glioblastoma | newly diagnosed | IV | 3 | 3 | WT | + | preserved | - | + | - | methylated | not done | N/A | TP53 I195T |
| 55 | 81 | F | Left Temporal | Glioblastoma | newly diagnosed | IV | 3 | 1.5 | WT | + | lost | - | + | - | Methylated | NO MATCH | <0.3 | TP53 H179Y |
| 56 | 48 | F | Right Frontal | Glioblastoma | newly diagnosed | IV | 4 | N/A | WT | not done | preserved | - | + | - | Methylated | Glioblastoma IDH wildtype | 0.99 | PIK3CA H1047L |
| 57 | 82 | M | Left Temporal | Glioblastoma | newly diagnosed | IV | 4 | 1 | WT | Scattered + | preserved | - | - | - | Unmethylated | control tissue inflammatory tumor microenvironment | 0.95 | BRAF G469V |
| 58 | 68 | M | Right Temporal | Glioblastoma | recurrent | IV | 4 | 2 | WT | Scattered + | not done | - | - | - | Unmethylated | Glioblastoma IDH wildtype | 0.66 | None |
| 59 | 29 | M | Left, Unspecified | Glioblastoma | recurrent | IV | 4 | N/A | WT | Focal + | lost | - | - | not done | Methylated | (anaplastic) pleomorphic xanthoastrocytoma | 0.54 | APC D1297N; JAK3 D730N; PIK3CA P458S |
| 60 | 76 | F | Right, Unspecified | Glioblastoma | newly diagnosed | IV | 3 | N/A | WT | - | preserved | - | - | not done | Unmethylated | Glioblastoma IDH wildtype | 0.99 | BRAF G596R |
| 61 | 55 | M | Right Parietal | Glioblastoma | newly diagnosed | IV | 1.5 | 1.5 | MUT | - | preserved | - | - | - | Unmethylated | not done | N/A | IDH1 R132C; PTPN11 R498W; TP53 R342Term; TP53 R273C |
| 62 | 37 | F | Right Frontal | Glioblastoma | newly diagnosed | IV | 2.5 | 0.5 | MUT | + | not done | - | + | - | Methylated | not done | N/A | not done |
| 63 | 44 | M | Left Temporal | Glioblastoma | newly diagnosed | IV | 2 | 1.5 | MUT | - | lost | not done | - | - | Unmethylated | Glioma IDH mutant | 0.98 | MET L829Q; TP53 R273C. |
| 64 | 49 | F | Right Parietal | Glioblastoma | recurrent | IV | 3.5 | 0.5 | MUT | + | preserved | - | + | not done | Methylated | Glioma IDH mutant | 0.99 | IDH1 R132H; TP53 R273C; TP53 R213Term |
| 65 | 31 | F | Left Temporal | Glioblastoma | newly diagnosed | IV | 3.5 | 1 | MUT | - | lost | - | + | not done | Unmethylated | Glioma IDH mutant | 0.99 | IDH1 R132G; JAK3 V722I |
| 66 | 35 | M | Left Temporal | Glioblastoma | recurrent | IV | 3.5 | 2 | MUT | + | lost | - | + | - | Methylated | Glioma IDH mutant | 0.99 | IDH1 R132H |
| 67 | 61 | M | Left, Unspecified | Glioblastoma | recurrent | IV | 3.5 | N/A | MUT | - | not done | - | + | + | Methylated | Glioma IDH mutant | 0.98 | IDH1 R132H; MLH1 A395T; PIK3CA H1047R; CDKN2A G55D; ATM N2736T, TP53 R306Term |
| **Normal subjects** | | | | | | | | | | | | | | | | | | |
| 1 | 46 | F | Whole Brain | Autopsy, Alzheimer's disease | | | 0 | N/A | not done | not done |  | not done | not done | not done | not done | not done | N/A | not done |
| 2 | 66 | M | Whole Brain | Autopsy, Hemorrhage (basal ganglia), Alzheimer's disease | | | 0 | N/A | not done | not done |  | not done | not done | not done | not done | not done | N/A | not done |
| 3 | 49 | M | Whole Brain | Autopsy, Hypertensive changes | | | 0 | N/A | not done | not done |  | not done | not done | not done | not done | not done | N/A | not done |
| 4 | 66 | F | Whole Brain | Autopsy, Hypoxic Injury, Alzheimer's disease) | | | 0 | N/A | not done | not done |  | not done | not done | not done | not done | not done | N/A | not done |
| 5 | 69 | M | Whole Brain | Autopsy, Hypoxic-ischemic changes, hydrocephalus *ex vacuo* | | | 0 | N/A | not done | not done |  | not done | not done | not done | not done | not done | N/A | not done |
| 6 | 38 | M | Right Temporal | Cortical Dysplasia, Focal Intractable epilepsy | | | 0 | N/A | WT | not done |  | not done | not done | not done | not done | not done | N/A | not done |
